# Responses of B-type natriuretic peptide (BNP), mature BNP and proBNP to sacubitril/valsartan differs between responders and non-responders

**DOI:** 10.1136/openhrt-2024-002990

**Published:** 2025-02-22

**Authors:** Toshio Nishikimi, Yasuaki Nakagawa, Shoichi Miyamoto, Takahiko Kanamori, Hideaki Inazumi, Hiromu Yanagisawa, Kenji Moriuchi, Hideaki Kinoshita, Yusuke Tamamura, Hiroyuki Takahama, Naoto Minamino, Koh Ono

**Affiliations:** 1 Cardiovascular Medicine, Kyoto University Graduate School of Medicine, Kyoto, Japan; 2 Internal Medicine, Wakakusa-Tatsuma Rehabilitation Hospital, Daito, Osaka, Japan; 3 Preventive Medicine and Division of Cardiovascular Medicine, KITANO HOSPITAL PIIF Tazuke-kofukai, Osaka, Japan; 4 Cardiology, Nishinomiya Watanabe Hospital, Nishinomiya, Hyogo, Japan; 5 Cardiovascular Medicine, National Cerebral and Cardiovascular Center, Suita, Osaka, Japan; 6 Rehabilitation, Wakakusa-Tatsuma Rehabilitation Hospital, Daito, Osaka, Japan; 7 Cardiovascular Medicine, Tohoku University Graduate School of Medicine, Sendai, Miyagi, Japan; 8 Biochemistry, National Cerebral and Cardiovascular Center, Suita, Osaka, Japan

**Keywords:** heart failure, heart failure, diastolic, heart failure, systolic, pharmacology, clinical, biomarkers

## Abstract

**Background:**

Earlier studies showed that measured changes in plasma B-type natriuretic peptide (BNP) levels are inconsistent after sacubitril/valsartan administration. The reason remains unknown but may reflect the fact that BNP immunoreactivity measured with commercial BNP assays (BNPcom) includes both mature BNP and proBNP, and neprilysin degrades only mature BNP. In addition, the responsiveness to sacubitril/valsartan varies among patients with heart failure. We investigated the mechanism underlying the inconsistency of BNP measurements after sacubitril/valsartan.

**Methods:**

We measured plasma mature BNP, proBNP and total BNP (mature BNP+proBNP) levels with our immunochemiluminescent assay as well as NT-proBNP, A-type natriuretic peptide (ANP) and BNPcom with conventional assays in 54 patients with heart failure, before (baseline) and after 2, 4, 8 and 12 weeks of sacubitril/valsartan administration. Responders were defined as having NT-proBNP levels at <70% of baseline after 12 weeks.

**Results:**

Among all patients, total BNP and BNPcom did not change with sacubitril/valsartan treatment, whereas NT-proBNP and proBNP decreased, mature BNP modestly increased and ANP greatly increased. Responders (n=31) exhibited smaller %changes in all natriuretic peptide levels than non-responders (n=23; all p<0.01). Receiver operating characteristic curves analysis to assess the ability of the %change in each natriuretic peptide at 4 weeks to detect responders showed that the area under the curve was about 0.80 for each peptide. There were good correlations between plasma natriuretic peptides levels at baseline and throughout the sacubitril/valsartan administration.

**Conclusion:**

These results suggest that the magnitude and direction of change in each BNP form depends on its substrate specificity for neprilysin, that differences in plasma levels of each BNP form between responders and non-responders appear early and persist and that BNPcom levels at 4 weeks can be applicable to prediction of the responders. Notably, our findings show that the idea that BNPcom cannot be used as a marker of heart failure after sacubitril/valsartan should be reconsidered.

**WHAT IS ALREADY KNOWN ON THIS TOPIC**

- Observed changes in plasma B-type natriuretic peptide (BNP) levels after administration of the angiotensin receptor-neprilysin inhibitor sacubitril/valsartan (Sac/Val) are inconsistent across studies, and the reason remains unknown.

**WHAT THIS STUDY ADDS**

- BNP immunoreactivity measured with commercial kits (BNPcom) includes both mature BNP and proBNP, a precursor of mature BNP.
- Neprilysin degrades mature BNP but not proBNP.
- With Sac/Val treatment, mature BNP levels increase slightly while proBNP levels decrease and, as a result, BNPcom does not change.
- In responders to Sac/Val, measured BNPcom decreases whereas in non-responders it increases.
- A-type natriuretic peptide increases much more than mature BNP after Sac/Val, but the magnitude of the change is smaller in responders than non-responders.
- BNPcom levels after 4 weeks of Sac/Val treatment can be used to detect responders with an area under the curve of 80%.

**HOW THIS STUDY MIGHT AFFECT RESEARCH, PRACTICE OR POLICY**

- This study will enable clinicians to easily interpret changes in BNPcom levels after Sac/Val.
- Importantly, this study shows that the idea that ‘BNPcom cannot be used as a marker of heart failure after Sac/Val treatment’ should be reconsidered.

## Introduction

 Recent studies have shown that the angiotensin receptor-neprilysin inhibitor, sacubitril/valsartan (Sac/Val), improves outcomes in patients with heart failure with preserved or diminished left ventricular function.^1^,^4^ The beneficial effects of Sac/Val have been attributed in part to inhibition of neprilysin, which leads to increases in various peptides, including A-type natriuretic peptide (ANP), B-type natriuretic peptide (BNP) and C-type natriuretic peptide (CNP).^5 6^ Current guidelines recommend that BNP be measured when making a diagnosis of heart failure, judging its severity or estimating a patient’s prognosis.^7 8^ However, one study has suggested that BNP should not be used as a marker for heart failure in patients taking Sac/Val because plasma BNP levels will likely be increased due to the drug-induced neprilysin inhibition.^9^ On the other hand, because BNP is not as good a substrate for neprilysin as ANP or CNP,^6 10^ the rise in BNP after neprilysin inhibition is actually small.^11^ Indeed, the results of previous studies indicate that after Sac/Val treatment plasma BNP levels may be increased,^12^ unchanged^5 13^ or decreased.^14^ The reason for the inconsistency in measured BNP levels remains uncertain.

One likely reason for the variation in plasma BNP levels during Sac/Val treatment is patient heterogeneity. A subanalysis of the Prospective Comparison of ARNI with ACEI to Determine Impact on Global Mortality and Morbidity in Heart Failure (PARADIGM-HF) study showed that patients with a large decreases in NT-proBNP after Sac/Val exhibit modest decreases in BNP and good prognoses, while patients with modest increases in NT-proBNP after Sac/Val treatment exhibit large increases in BNP after Sac/Val and poor prognoses.^12^ In other words, there may be large differences in BNP responses between responders and non-responders to Sac/Val. In the present study, therefore, we analysed natriuretic peptide (NP) measurements after separating responders and non-responders.

Another reason for the inconsistent results may be the assay system used to measure BNP. BNP immunoassays currently used in clinical settings employ the sandwich method, which entails use of two antibodies, one for capture and the other for detection.^15^ However, these BNP assay systems currently in use may differently cross-react with the precursor proBNP, considerable amounts of which circulate in human blood.^16^,^18^ This may lead to variation in BNP measured with commercial kits (BNPcom) after Sac/Val treatment,^19^ as mature BNP is subject to degradation by neprilysin, while proBNP is not.^20^ Moreover, the contribution of proBNP to the overall BNP immunoreactivity varies depending on the patient’s condition, and there are no reports in which plasma levels of proBNP and mature BNP before and after Sac/Val administration were measured.

We previously developed a new chemiluminescence immunoassay for proBNP and total BNP (mature BNP+proBNP) that enables accurate calculation of mature BNP, the proBNP/total BNP ratio and the mature BNP/total BNP ratio in patients with heart failure.^21^ Using this system, we found that the proBNP/total BNP ratio varies depending on the pathophysiology of the heart failure.^22^,^24^ In the present study, we used this method to measure proBNP, total BNP and mature BNP before and after 2, 4, 8 and 12 weeks of Sac/Val administration. In addition, we measured NT-proBNP, ANP and BNPcom using commercially available assays. Our findings shed new light on the mechanism underlying the variation in measured BNP levels after Sac/Val administration.

## Methods

### Study design

This was a prospective observational study of patients who were hospitalised or followed by outpatient clinics at the Kyoto University Hospital (Kyoto), Wakakusa-Tatsuma Rehabilitation Hospital (Osaka) and Watanabe Hospital (Hyogo) of Japan.

### Study population

The eligibility criteria were as follows: (1) age from 20 to 85 years, (2) patients stabilised after treatment for acute heart failure, (3) patients with chronic heart failure followed at an outpatient clinic and (4) patients who provided written informed consent to participate. Patients on dialysis or diagnosed with acute coronary syndrome were excluded. Between November 2021 and March 2023, we obtained written informed consent from a total of 64 patients with heart failure (aged 32–89 years). Blood samples for measurement of plasma NP levels were collected before (baseline) and after 2, 4, 8 and 12 weeks of Sac/Val treatment. We excluded 10 patients who did not undergo at least four blood tests. Data on the remaining 54 patients were analysed.

### Measurement of mature BNP, proBNP, total BNP, NT-proBNP, ANP and BNP

Blood samples were collected in plastic tubes containing EDTA (1.5 mg/mL) and aprotinin (500 kallikrein inhibitor units/mL). Plasma was separated and stored at −80°C before measurement. Plasma proBNP and total BNP were measured using the chemiluminescent immunoassays developed in our laboratory.^21^ Since proBNP and mature BNP are recognised at the almost same avidity in the total BNP assay, mature BNP was calculated using the following equation: mature BNP=(total BNP–proBNP). Serum NT-proBNP levels were measured using an Elecsys proBNP II assay system (Roche Diagnostics, Basel, Switzerland). Plasma ANP and BNPcom levels were measured using CL AIA-PACK ANP and CL AIA-PACK BNP kits (Tosoh), respectively. We have shown a model diagram of each BNP molecular form and its assay system (online supplemental figure 1).

### Echocardiography

Echocardiography was routinely performed before starting treatment and after 12 weeks of Sac/Val treatment in patients with heart failure. Left ventricular end-diastolic volume index (LVEDVI), left ventricular end-systolic volume index and left ventricular ejection fraction (LVEF) were measured using the modified Simpson’s method.

### Definition of responders to Sac/Val

The study participants were divided into two groups: Sac/Val responders or non-responders. Responders were defined as patients with NT-proBNP levels at <70% of their baseline values after 12 weeks of Sac/Val treatment, while non-responders were defined as patients other than those defined above.^25 26^

### Sample size estimation for responder versus non-responder comparison and the sample size for the receiver operating characteristic curves

In our previous study,^27^ the SD of the serum N-terminal proBNP levels of the patient with heart failure with New York Heart Association (NYHA) class II was 831 pg/mL. Because the true difference between the means of the responder group and the means of the non-responder group is 730,^28^ it is necessary to study 19 participants per group to be able to reject the null hypothesis that the population means of the groups are equal at a statistical power of 0.8 and a type I error rate of 0.05. Regarding sample size for receiver operating characteristic (ROC) analysis, we used an online sample size calculation tool (https://sample-size.net/sample-size-ci-for-auroc/) to estimate the required sample size, assuming an area under the receiver operating characteristic of 0.90, a proportion of responder of 0.60, a CI width of 0.20 and a confidence level of 0.95 from our preliminary study. The required sample size was estimated to be 37. Therefore, we planned to enrol >50 patients with heart failure (mainly NYHA II).

### Statistical analysis

Continuous data are presented as the mean±SD, and non-parametric data as the median (IQR 25–75 percentile). Statistical differences between the two groups were analysed using Student’s t-test or the Mann-Whitney U test, as appropriate. Categorical data were expressed as incidences and percentages, and comparisons were made using the χ^2^ test. Correlation coefficients were calculated using linear regression analysis. The time-course change from baseline to the treatment period was assessed with Friedman’s test. The relationships between the %changes from baseline for each NP after 4 weeks of Sac/Val and responders defined at 12 weeks were assessed using ROC curves, and differences in diagnostic performance were compared based on the area under the curve (AUCs). Values of p<0.05 were considered statistically significant. Statistical analyses were performed using SPSS V.28.0 (IBM, Armonk, New York, USA).

## Results

Table 1 shows the clinical characteristics of the patients. In brief, the study included 54 subjects >20 years of age who were hospitalised for an episode of acute decompensated heart failure or followed at an outpatient clinic. These patients were classified as NYHA functional class I to III with a mean LVEF of 48% and blood pressure >100 mm Hg at baseline.

**Table 1:** Clinical characteristics of all patients with HF receiving Sac/Val

Characteristics	Baseline
Number	54
Age, years	71±16
Male, n (%)	26 (48.1)
Heart rate, beats/min	76±13
Systolic blood pressure, mm Hg	129±18
LV ejection fraction, %	48.0±12.7
LV ejection fraction <40, n (%)	17 (31.5)
LV ejection fraction 40~49, n (%)	13 (24.1)
LV ejection fraction >50, n (%)	24 (44.4)
Pre-LVEDVI, mL/m^2^	53.3 (46.1–71.3)
Pre-LVESVI, mL/m^2^	27.6 (21.5–41.5)
Body mass index, kg/m^2^	22.0±3.8
Pre-eGFR, mL/min	54.4±20.6
Chronic af, n (%)	8 (14.8)
HT, n (%)	39 (72.2)
DM, n (%)	19 (35.2)
Ischaemic heart disease, n (%)	13 (24.1)
De novo HF, n (%)	23 (42.6)
ACEI/ARB, n (%)	36 (66.6)
β-Blocker, n (%)	30 (55.6)
MRA, n (%)	19 (35.2)
Diuretic, n (%)	24 (44.4)
NYHA	
I, n (%)	18 (33.3)
II, n (%)	29 (53.7)
III, n (%)	7 (13.0)
Sac/Val final dose	
24/26 mg, n (%)	5 (9.3)
49/51 mg, n (%)	19 (35.2)
97/103 mg, n (%)	16 (29.6)
194/206 mg, n (%)	14 (25.9)

Value are means±SD or median (Q1 – Q3).

ACEI, ACE inhibitor; af, atrial fibrillation; ARB, angiotensin receptor blocker; DM, diabetes mellitus; eGFRestimated glomerular filtration rateESVI, end-systolic volume index; HFheart failureHT, hypertension; LV, left ventriclular; LVEDVI, left ventricular end-diastolic volume index; LVESVIleft ventricular end-systolic volume indexMRA, mineralocorticoid receptor antagonist; NYHA, New York Heart Association; Sac/Val, sacubitril/valsartan

**Figure 1:**
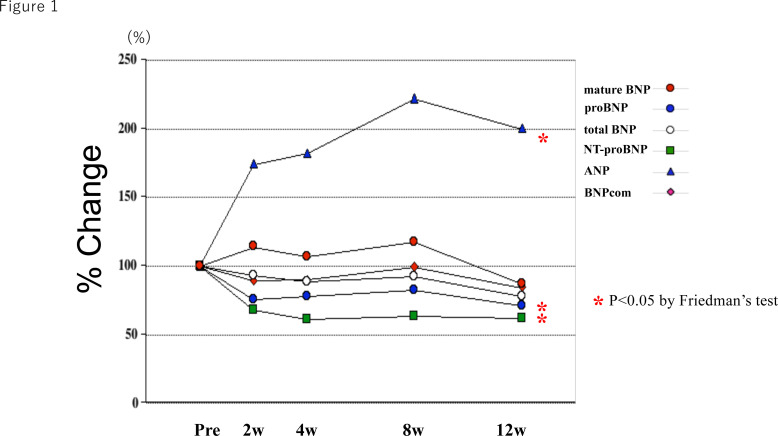
Per cent changes in mature BNP, proBNP, total BNP, NT-proBNP, ANP and BNPcom during 12 weeks of sacubitril/valsartan treatment. The red circles, blue circles, white circles, green squares, blue triangles and red diamonds, respectively, represent the median values of the ratios of mature BNP, proBNP, total BNP, NT-proBNP, ANP and BNPcom levels to the baseline level after the indicated weeks of treatment. ANP, A-type natriuretic peptide; BNP, B-type natriuretic peptide; BNPcom, commercial BNP.

Plasma levels of mature BNP, proBNP, total BNP, NT-proBNP, ANP and BNPcom at baseline and after 2–12 weeks of Sac/Val treatment are shown in table 2. Plasma levels of mature BNP, total BNP and BNPcom did not change during the Sac/Val treatment, whereas proBNP and NT-proBNP decreased and ANP increased. For the total study population, the %changes from baseline for all NPs after 2, 4, 8 and 12 weeks of Sac/Val treatment are presented in figure 1. Both ANP and mature BNP are substrates for neprilysin, but while ANP increased significantly after Sac/Val administration, increases in mature BNP did not reach statistical significance. NT-proBNP and proBNP, which are not substrates for neprilysin, were both significantly reduced after Sac/Val administration, although the effect on NT-proBNP was greater.

**Table 2:** Plasma levels of mature BNP, proBNP, total BNP, NT-proBNP, ANP and BNPom at baseline and after 2, 4, 8 and 12 weeks of sacubitril/valsartan in the responders and non-responders

Variables	Total (n=54)	Responder (n=31)	Non-responder (n=23)	P value
Mature BNP baseline (pmol/L)	12.5 (3.7–25.5)	16.2 (4.4–35.9）	7.8 (1.9–18.2)	0.3020
Mature BNP week 2 (pmol/L)	12.9 (3.1–25.1)	11.2 (3.7–38.3)	13.0 (2.0–23.8)	0.8543
Mature BNP week 4 (pmol/L)	11.9 (3.4–32.9)	11.7 (4.7–48.1)	12.1 (2.5–39.3)	0.9930
Mature BNP week 8 (pmol/L)	13.7 (5.8–39.2)	12.1 (2.4–45.0)	16.4 (7.3–31.9)	0.0992
Mature BNP week 12 (pmol/L)	13.3 (2.8–28.4)	11.0 (2.6–27.8)*	17.1 (4.6–31.1)*	0.9926
proBNP baseline (pmol/L)	17.4 (6.9–36.9)	24.1 (8.2–49.7)	10.3 (2.5–22.4)	0.0257
proBNP week 2 (pmol/L)	10.4 (4.1–23.8)	10.1 (4.2–26.0)	10.6 (2.5–27.0)	0.5462
proBNP week 4 (pmol/L)	11.3 (4.3–30.9)	11.7 (4.3–30.9)	11.2 (3.0–40.4)	0.8337
proBNP week 8 (pmol/L)	13.0 (6.5–31.0)	15.6 (6.2–28.4)	13.2 (8.2–32.0)	0.9089
proBNP week 12 (pmol/L)	9.4 (4.7–20.7)*	8.8 (4.4–17.1)*	11.3 (4.9–25.6)	0.4587
Total BNP baseline (pmol/L)	31.2 (11.7–68.1)	42.6 (13.0–97.2)	19.1 (3.7–37.3)	0.0307
Total BNP week 2 (pmol/L)	22.6 (7.3 (53.5)	18.6 (8.2–76.9)	23.0 (4.5–53.5)	0.5232
Total BNP week 4 (pmol/L)	24.6 (9.4–57.3)	24.0 (9.4–80.3)	25.2 (5.4–86.0)	0.7861
Total BNP week 8 (pmol/L)	32.4 (12.3–68.0)	31.9 (9.4–70.9)	32.4 (13.5–68.0)	0.6292
Total BNP week 12 (pmol/L)	26.3 (7.5–38.8)	23.2 (5.2–42.9)*	27.8 (8.4–50.7)*	0.3841
NT-proBNP baseline (pg/mL)	857 (243–2220)	1190 (645–2670)	315 (187–1720)	0.0121
NT-proBNP week 2 (pg/mL)	451 (167–1260)	503 (181–1710)	264 (106–1210)	0.1752
NT-proBNP week 4 (pg/mL)	431 (223–1290)	551 (226–1370)	287 (110–1330)	0.4896
NT-proBNP week 8 (pg/mL)	513 (253–1270)	429 (234–1055)	735 (253–1270)	0.4616
NT-proBNP week 12 (pg/mL)	378 (194–1345)*	364 (208–1200)*	531 (190–1380)	0.8385
ANP baseline (pg/mL)	53 (30–120)	60 (30–155)	46 (25–108)	0.2308
ANP week 2 (pg/mL)	100 (47–183)	99 (46–192)	101 (69–186)	0.2447
ANP week 4 (pg/mL)	110 (60–228)	106 (46–240)	116 (73–482)	0.1520
ANP week 8 (pg/mL)	112 (68–232)	74 (46–197)	172 (100–242)	0.0455
ANP week 12 (pg/mL)	117 (54–215)*	80 (47–167)	145 (87–235)*	0.0407
BNPcom baseline (pg/mL)	126 (57–275)	188 (76–429)	72 (40–243)	0.0411
BNPcom week 2 (pg/mL)	107 (35–260)	131 (47–278)	92 (22–271)	0.7997
BNPcom week 4 (pg/mL)	105 (33–254)	113 (33–242)	99 (26–331)	0.3805
BNPcom week 8 (pg/mL)	136 (60–271)	122 (52–208)	140 (65–318)	0.3805
BNPcom week 12 (pg/mL)	101 (40–210)	94 (41–183)*	120 (39–291)*	0.2911

Values are median (Q1-–Q3). Table dData show concentrations of mature (mature BNP), proBNP, total BNP, N-terminal pro- (NT-proBNP), (ANP) and BNPcom at baseline (n=54), week 2 (n=54), week 4 (n=54), week 8 (n=42), and week 12 (n=52).

* PP<0.05. Time course change were assessed by Friedman’s test. P value compares the responder group towith the non-responder group.

ANPatrial natriuretic peptideBNPB-type natriuretic peptideBNPcomcommercial BNPNT-proBNPN-terminal pro-B-type natriuretic peptide

The clinical characteristics of the patients after separating the responders and non-responders are shown in table 3. Age, gender, heart rate, systolic blood pressure, body mass index, renal function, NYHA class and the presence of comorbidities such as hypertension, diabetes mellitus and ischaemic heart disease were similar between the two groups. However, the responders had lower baseline LVEFs, greater ΔLVEFs and ΔLVEDVIs, and more de novo heart failure than the non-responders. There was no difference between the two groups with respect to their drug prescriptions or final dose of Sac/Val.

**Table 3:** Clinical characteristics of patients with heart failure with sacubitril/valsartan: responders versus non-responders

Variables	Non-responder (n=23)	Responder (n=31)	P value
Age, years	74±15	69±17	0.263
Male, n (%)	10 (45.5)	16 (50.0)	0.743
Heart rate, beats/min	75±11	77±14	0.685
Systolic blood pressure, mm Hg	125±17	132±19	0.172
Pre-LV ejection fraction, %	53.4±10.4	44.3±13.0	0.012
Pre-LV ejection fraction <40, n (%)	4 (17.3)	13 (41.9)	0.142
Pre-LV ejection fraction 40–49, n (%)	6 (26.1)	7 (22.6)	
Pre-LV ejection fraction >50, n (%)	13 (56.5)	11 (35.4)	
Post-LV ejection fraction, %	54.9±12.6	54.6±11.6	
Post-LV ejection fraction <40, n (%)	3 (13.0)	3 (9.7)	0.829
Post-LV ejection fraction 40–49, n (%)	7 (30.4)	8 (25.8)	
Post-LV ejection fraction >50, n (%)	13 (56.5)	20 (54.5)	
ΔLVEF, %	1.5±6.9	10.7±9.8	0.011
Pre-LVEDVI, mL/m^2^	52.5 (45.5–62.7)	62.4 (47.6–82.3)	0.170
Post-LVEDVI, mL/m^2^	49.7 (44.4–59.1)	50.8 (43.7–67.0)	0.819
ΔLVEDVI, mL/m^2^	2.0 (−3.1–5.0)	−4.6 (−18.0–−0.5)	0.016
Pre-LVESVI, mL/m^2^	24.2 (18.7–31.6)	29.0 (22.8–49.8)	0.091
Post-LVESVI, mL/m^2^	24.9 (16.1–32.7)	21.8 (16.9–31.6)	0.993
ΔLVESVI, mL/m^2^	−3.8 (−8.0–1.5)	−4.9 (−9.6–−1.6)	0.295
Body mass index, kg/m^2^	21.7±3.9	22.3±3.7	0.812
eGFR, mL/min	54.7±22.3	54.3±19.7	0.805
Chronic af, n (%)	5 (22.7)	3 (9.4)	0.175
HT, n (%)	15 (68.2)	24 (75.0)	0.583
DM, n (%)	9 (40.9)	10 (31.3)	0.465
Ischaemic heart disease, n (%)	4 (18.2)	9 (28.1)	0.401
De novo HF, n (%)	4 (18.2)	19 (59.4)	0.003
ACEI/ARB, n (%)	15 (65.2)	21 (67.7)	0.846
β-Blocker, n (%)	14 (63.6)	16 (50.0)	0.322
MRA, n (%)	5 (22.7)	14 (43.8)	0.112
Diuretic, n (%)	9 (40.9)	15 (46.9)	0.665
NYHA, n (%)			
I, n (%)	8 (36.4)	10 (31.3)	0.312
II, n (%)	13 (59.1)	16 (50.0)	
III, n (%)	1 (4.5)	6 (18.8)	
Sa/Val final dose			
24/26 mg, n (%)	1 (4.5)	4 (12.5)	0.689
49/51 mg, n (%)	8 (36.4)	11 (34.4)	
97/103 mg, n (%)	6 (27.3)	10 (31.3)	
194/206 mg, n (%)	7 (31.8)	7 (21.9)	

ACEIACE inhibitorafatrial fibrillationARBangiotensin receptor blockerDMdiabetes mellitusESVIleft ventricular end-systolic volume indexHThypertensionLVleft ventriclularLVEDVIleft ventricular end-diastolic volume indexLVEFleft ventricular ejection fractionMRAmineralocorticoid receptor antagonistNYHANew York Heart AssociationSac/Valsacubitril/valsartanΔdelta

Plasma levels of all NPs at baseline and after 2–12 weeks of Sac/Val in the two groups are shown in table 2. At baseline, levels of proBNP, total BNP, NT-proBNP and BNPcom were all significantly higher in the responders than non-responders, whereas there were no between-group differences in mature BNP or ANP levels at baseline. During Sac/Val treatment, mature BNP, total BNP, and BNPcom significantly deceased in responders, whereas they significantly increased in non-responders. On the other hand, proBNP and NT-proBNP significantly decreased in responders without changing them in non-responders. By contrast, after 8 and 12 weeks of Sac/Val treatment, plasma ANP levels were significantly higher in non-responders than responders.

**Figure 2:**
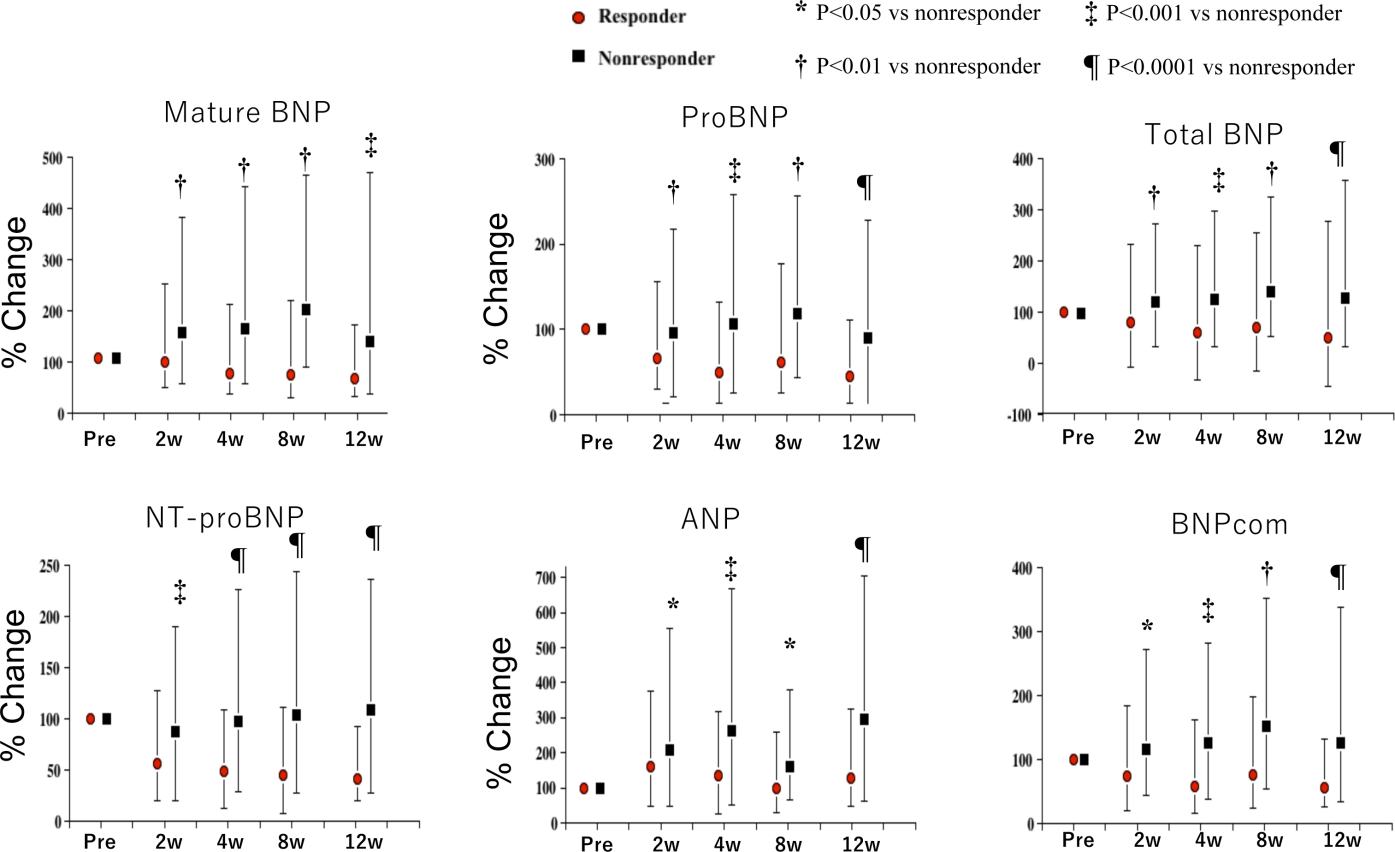
Percentage changes in mature BNP, proBNP, total BNP, NT-proBNP, ANP and BNPcom in responders and non-responders during 12 weeks of sacubitril/valsartan treatment. The red circles (responders) and black squares (non-responders) represent the median values and the whiskers quartiles 1 and 3. ANP, A-type natriuretic peptide; BNP, B-type natriuretic peptide; BNPcom, commercial BNP.

The %changes from baseline for each NP after 2, 4, 8 and 12 weeks of Sac/Val treatment in the two groups are presented in figure 2. At each measured time point, the %changes were all significantly smaller among the responders than non-responders. In addition, the correlation coefficients and corresponding p values between each NP at baseline and after 2, 4, 8 and 12 weeks of Sac/Val show good correlations among the different BNP forms (online supplemental table 1). Notably, there were modest correlations throughout the time-course between ANP, which is a good substrate for neprilysin, and NT-proBNP, which is not a substrate for neprilysin.

**Figure 3:**
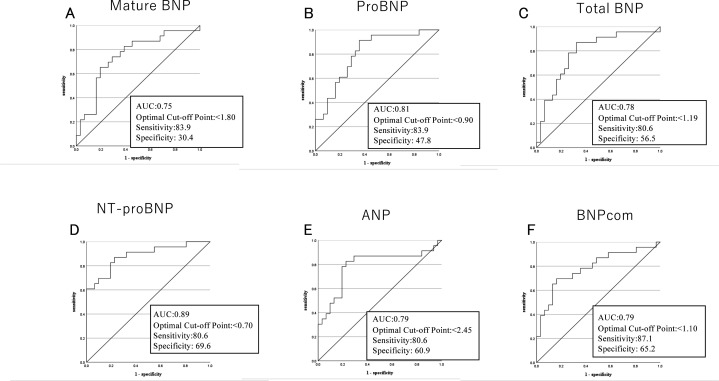
Receiver operating characteristic curve analyses to predict responders based on the %change from baseline in the concentration of each natriuretic peptide after 4 weeks of sacubitril/valsartan treatment. Area under the curves, optimal cut-off points, sensitivities and specificities are shown.

The ability of the %change from baseline in the plasma level of each NP after 4 weeks of Sac/Val to detect responders was assessed by constructing ROC curves (figure 3A–F). The AUCs for mature BNP, proBNP, total BNP, NT-proBNP, ANP and BNPcom at 4 weeks were 0.75, 0.81, 0.78, 0.89, 0.79 and 0.79, respectively (all p<0.001). The higher AUC for NT-proBNP is not surprising, as responders were defined based on NT-proBNP. However, it is noteworthy that the other NPs had similar AUCs, irrespective of their susceptibility to neprilysin inhibition. For responders, the sensitivity, specificity and optimal cut-point for the %change from baseline in each NP at 4 weeks is also shown in figure 3. Although the optimal NP concentration for detection of responders differed depending on the impact of neprilysin inhibition (ANP >2, proBNP and NT-proBNP <1 and total BNP, mature BNP and BNPcom <2), AUCs, sensitivity and specificity did not greatly differ among the molecules.

## Discussion

In this study, we separately measured mature BNP and proBNP, the two components of BNP immunoactivity measured with the current commercially available BNP assay systems. Measurements were made in patients with heart failure before and after Sac/Val administration, and the changes in these components were assessed. Among all patients, Sac/Val treatment had little effect on total BNP or BNPcom. On the other hand, ANP greatly increased and mature BNP increased to a smaller degree while proBNP and NT-proBNP declined. When we divided patients into responders and non-responders, the %change in all NPs was significantly smaller among the responders than non-responders throughout the entire Sac/Val treatment period. The correlation coefficients between these molecules at baseline and after 2, 4, 8 and 12 weeks of treatment ranged from modest to good. ROC curve analysis to assess whether the %change in each NP at 4 weeks from baseline was predictive of which patients would respond to Sac/Val treatment suggested that the area under the curve was about 0.80 for each NP. Thus, the magnitude and direction of change in each NP induced by Sac/Val depends on the peptide’s substrate specificity for neprilysin, and the differences between responders and non-responders appear early and persist.

The beneficial effects of Sac/Val on the outcomes of patients with heart failure may be attributable to its ability to inhibit degradation of ANP, BNP and CNP,^5 6 10^ the protective effects of which in heart failure are well known.^29^ Because Sac/Val inhibits mature BNP degradation, an earlier study suggested that when Sac/Val is used to treat heart failure, BNPcom should be elevated in all cases and therefore should not be used as a marker for heart failure.^9^ On the other hand, mature BNP is not a good substrate for neprilysin,^6 10 30^ and another study demonstrated that plasma BNPcom levels were actually less likely than, for example, ANP to be elevated by neprilysin inhibition in humans.^11^ In fact, plasma BNPcom levels are reported to be increased,^12^ unchanged^5 13^ or decreased^14^ after Sac/Val administration. One reason for this inconsistency is that the current commercially available immunoassays for BNP cross-react with proBNP, meaning that the measured BNPcom levels include mature BNP+proBNP.^21^ We have developed a new assay system to measure proBNP and total BNP, which we can use to accurately calculate levels of mature BNP.^21^ Using that assay to measure mature BNP, we found that ANP was greatly increased by Sac/Val treatment, but mature BNP was increased to a smaller degree. This is consistent with earlier studies showing the high substrate specificity of neprilysin for ANP but low specificity for BNP.^6 10 30^ ANP, like other NPs, has its plasma level primarily regulated by cardiac production, which is stimulated by cardiac loading. Since ANP is most susceptible to degradation by neprilysin, even if production decreases in responders, plasma ANP level may increase when the inhibition of degradation is greater than decrease of production. In non-responders, production increases, and because the inhibition of degradation by neprilysin is greater, it greatly increases. Levels of proBNP and NT-proBNP, which are not substrates of neprilysin, decreased.^20^ Nevertheless, significant correlations were observed between ANP and proBNP or NT-proBNP. This suggests that plasma NP levels are regulated mainly by cardiac production and that neprilysin inhibition is only a modifying factor. The previously observed variability in BNPcom levels after Sac/Val treatment may therefore reflect differences in the percentages of responders among the study participants.

The ability of the %change in each NP after 4 weeks of Sac/Val administration to predict responders was also examined. With ROC curve analysis, we observed that the AUC for NT-proBNP was higher than for other NPs. This was expected, given how we defined responders. It is noteworthy, however, that the AUCs for the other NPs were all nearly as high. Plasma ANP levels increased with Sac/Val,while proBNP levels decreased, but the AUCs for the %change after 4 weeks were similar between the two. This is again consistent with the idea that plasma levels of all NPs are primarily regulated by their cardiac production and that Sac/Val acts merely as a modifying factor. On the other hand, the optimal cut-off for the %change needed to distinguish responders from non-responders was comparatively high for ANP because plasma ANP levels are significantly increased by neprilysin inhibition. On the other hand, NT-proBNP and proBNP have lower optimal cut-off values because they are not degraded by neprilysin.^22^ The optimal cut-off values for BNPcom and total BNP were lower than the cut-off for ANP but higher than those for NT-proBNP and proBNP because their levels are modestly affected by neprilysin inhibition. Mature BNP is also weakly affected by neprilysin inhibition,^6 10^ so its optimal cut-off value was similar to those of BNPcom and total BNP. Thus, during Sac/Val treatment, the optimal cut-off for the %change in the plasma NP level needed to distinguish responders from non-responders reflects each NP’s susceptibility to degradation by neprilysin.

In this context, it should be remembered that overall NP production decreases (in responders) when cardiac wall stress is reduced, that NP production remains unchanged or increases (in non-responders) when cardiac wall stress is unchanged or increased and that neprilysin inhibition similarly affects NP levels in both responders and non-responders. Consequently, the difference in overall plasma NP levels between responders and non-responders will persist, even though the magnitude and direction of the difference largely depends on the substrate specificity of each NP for neprilysin.

This research has several limitations. First, the number of participants was small. If the number were increased, we would expect the impact on some significantly affected indicators to increase, but we do not think the basic fact of the changes in NP levels seen in this study would be affected. Second, mean LVEF was relatively high in this study, and participants included those with heart failure with preserved EF (HFpEF) and those with heart failure with reduced EF (HFrEF). We found that responders had lower LVEFs and higher BNP levels than non-responders. However, the purpose of this study was not to assess the effects of Sac/Val on outcomes in patients with heart failure, but rather to assess the effects of Sac/Val on the measured levels of each NP and its diagnostic significance in patients with heart failure. In the future, it would be a good idea to consider the effects of Sac/Val on each NP separately for patients with HFpEF or HFrEF.

In conclusion, the results of this study suggest (1) that the magnitude and direction of change in individual NPs depend on their substrate specificity for neprilysin, (2) that differences in plasma BNPcom levels between responders and non-responders appear early and persist and (3) that BNPcom levels measured after 4 weeks of Sac/Val treatment can be applicable to detect responders with AUC 80%. Importantly, the idea that 'BNP should not be used as a marker of heart failure after administration of Sac/Val' should therefore be reconsidered.

## supplementary material

10.1136/openhrt-2024-002990 online supplemental figure 1

10.1136/openhrt-2024-002990 online supplemental table 1

## Data Availability

All data relevant to the study are included in the article or uploaded as supplementary information.
