# Supplementary material for: Responses of B-type natriuretic peptide (BNP), mature BNP and proBNP to sacubitril/valsartan differs between responders and non-responders
Source: Open Heart. 2025 Feb 22;12(1):e002990. doi: 10.1136/openhrt-2024-002990 (PMC11848661; doi:10.1136/openhrt-2024-002990)

Supplemental Figure 1-A

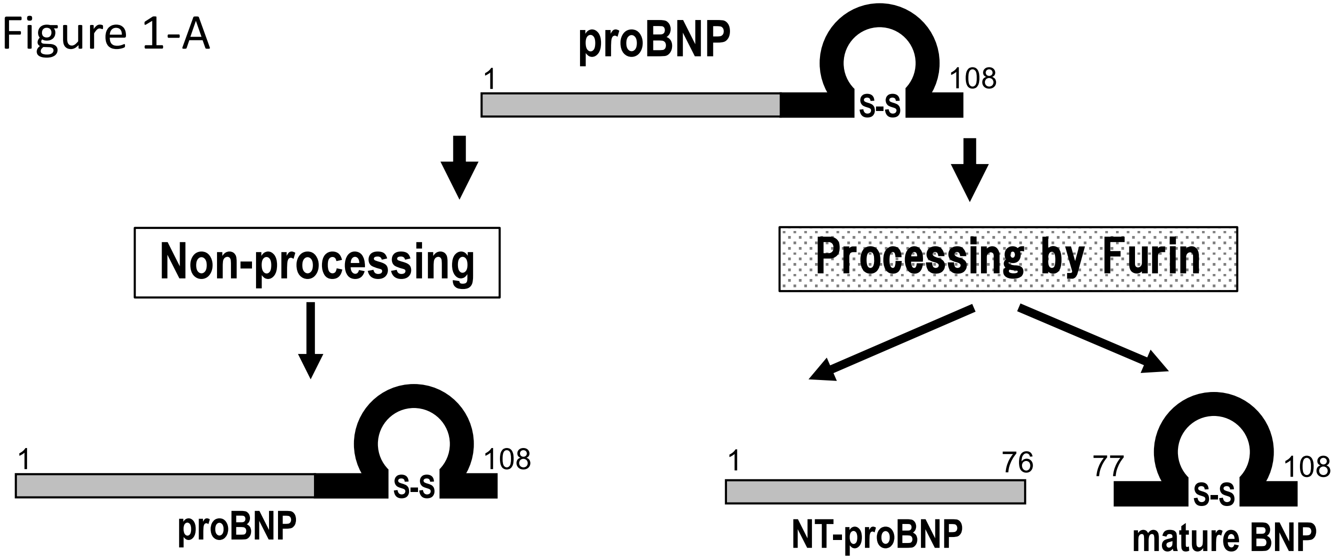

Supplemental Figure 1-B

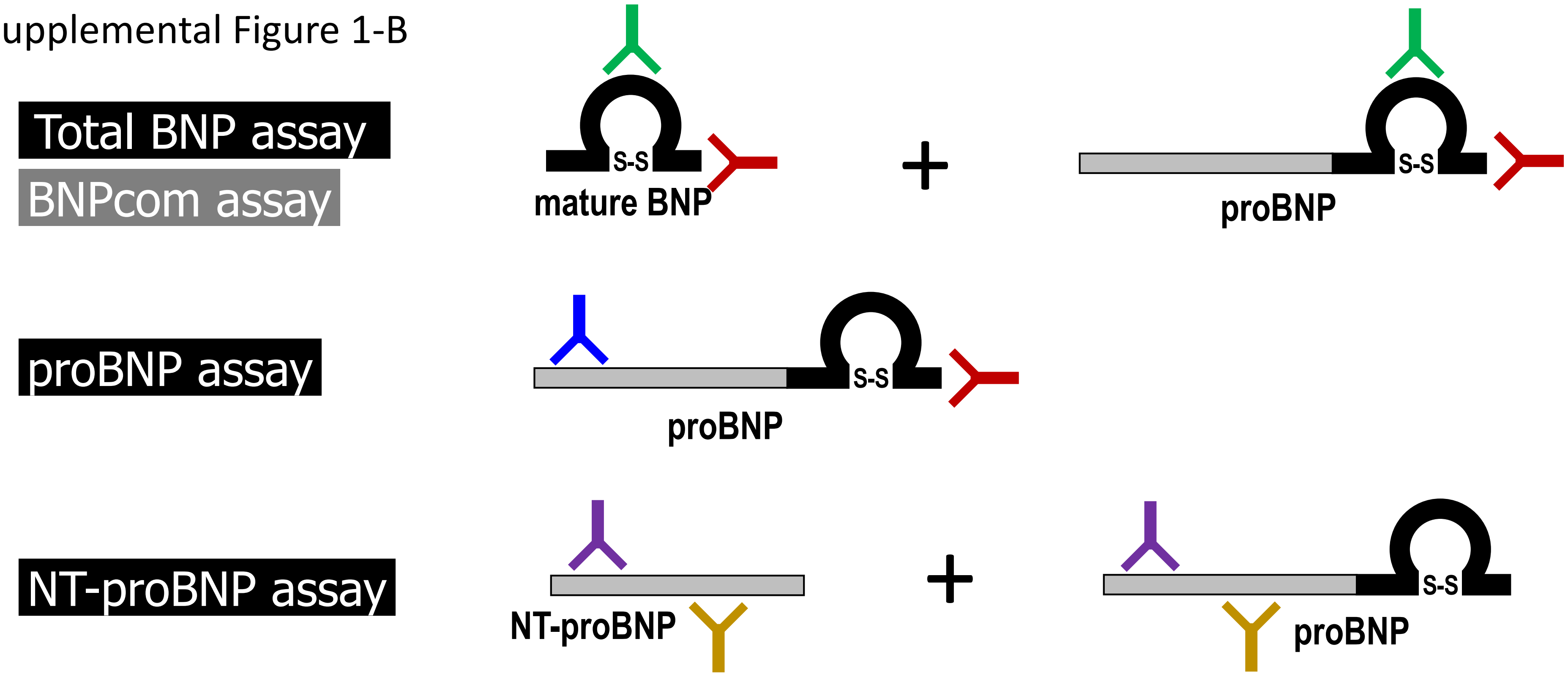

Supplement: online supplemental figure 1 [file openhrt-12-1-s001.pdf]
