# Supplementary material for: Responses of B-type natriuretic peptide (BNP), mature BNP and proBNP to sacubitril/valsartan differs between responders and non-responders
Source: Open Heart. 2025 Feb 22;12(1):e002990. doi: 10.1136/openhrt-2024-002990 (PMC11848661; doi:10.1136/openhrt-2024-002990)
Supplement: online supplemental table 1 [file openhrt-12-1-s002.pdf]

Supplementary Table 1. Correlation coefficients and p value between each peptide concentrations at baseline and 2week, 4week, 8week and 12 week after sacubitril/valsartan

	Baseline		2 week		4 week		8 week		12 week	
**Variables**	**Correlation coefficient**	**P value**	**Correlation coefficient**	**P value**	**Correlation coefficient**	**P value**	**Correlation coefficient**	**P value**	**Correlation coefficient**	**P value**
**mature BNP vs proBNP**	r= 0.868	P < 0.0001	r= 0.840	P < 0.0001	r= 0.897	P < 0.0001	r= 0.811	P < 0.0001	r= 0.734	P < 0.0001
**mature BNP vs total BNP**	r= 0.968	P < 0.0001	r= 0.981	P < 0.0001	r= 0.984	P < 0.0001	r= 0.971	P < 0.0001	r= 0.959	P < 0.0001
**mature BNP vs NT-BNP**	r= 0.697	P < 0.0001	r= 0.680	P < 0.0001	r= 0.687	P < 0.0001	r= 0.346	P = 0.0243	r= 0.582	P = 0.0113
**mature BNP vs ANP**	r= 0.624	P < 0.0001	r= 0.325	P = 0.0159	r= 0.470	P = 0.0003	r= 0.459	P = 0.0019	r= 0.884	P < 0.0001
**mature BNP vs BNPcom**	r= 0.756	P < 0.0001	r= 0.938	P < 0.0001	r= 0.798	P < 0.0001	r= 0.834	P < 0.0001	r= 0.910	P < 0.0001
**proBNP vs total BNP**	r= 0.965	P < 0.0001	r= 0.931	P < 0.0001	r= 0.962	P < 0.0001	r= 0.926	P < 0.0001	r= 0.896	P < 0.0001
**proBNP vs NT-BNP**	r= 0.647	P < 0.0001	r= 0.792	P < 0.0001	r= 0.740	P < 0.0001	r= 0.618	P < 0.0001	r= 0.663	P < 0.0001
**proBNP vs ANP**	r= 0.406	P = 0.0021	r= 0.343	P = 0.0107	r= 0.428	P = 0.0011	r= 0.648	P < 0.0001	r= 0.456	P = 0.0006
**proBNP vs BNPcom**	r= 0.716	P < 0.0001	r= 0.890	P < 0.0001	r= 0.769	P < 0.0001	r= 0.842	P < 0.0001	r= 0.595	P < 0.0001
**total BNP vs NT-BNP**	r= 0.695	P < 0.0001	r= 0.747	P < 0.0001	r= 0.726	P < 0.0001	r= 0.471	P = 0.0014	r= 0.656	P < 0.0001
**total BNP vs ANP**	r= 0.535	P < 0.0001	r= 0.344	P = 0.0104	r= 0.465	P = 0.0003	r= 0.557	P < 0.0001	r= 0.768	P < 0.0001
**total BNP vs BNPcom**	r= 0.762	P < 0.0001	r= 0.956	P < 0.0001	r= 0.807	P < 0.0001	r= 0.878	P < 0.0001	r= 0.843	P < 0.0001
**NT-BNP vs ANP**	r= 0.625	P < 0.0001	r= 0.530	P < 0.0001	r= 0.558	P < 0.0001	r= 0.821	P < 0.0001	r= 0.516	P < 0.0001
**NT-BNP vs BNPcom**	r= 0.691	P < 0.0001	r= 0.747	P < 0.0001	r= 0.710	P < 0.0001	r= 0.567	P < 0.0001	r= 0.560	P < 0.0001
**ANP vs BNPcom**	r= 0.644	P < 0.0001	r= 0.435	P = 0.0009	r= 0.563	P < 0.0001	r= 0.574	P < 0.0001	r= 0.920	P < 0.0001

BNP, B-type natriuretic peptide; proBNP, proB-type natriuretic peptide; ANP, A-type natriuretic peptide; NT-BNP, N-terminal proBNP; BNPcom, BNP measured by commercial BNP assay
